# The Jaeger Revolution

**Published:** 1888-04-21

**Authors:** 


### THE JAEGER REVOLUTION.

There seems no doubt that the new ideas as to clothing and bedding which were promulgated a few years since by Dr. G. Jaeger, of Stuttgart, have taken extensive root in this country, and, as our American cousins put it, have "come to stay."

At first laughed at as a fad, or as an ingenious mode of advertising, the Jaeger Sanitary Woollen System has effected an important change in the lives of a large number of English people, and the interest felt in the subject waxes rather than wanes. It is not difficult to trace the reason for this endurance of interest in a question which, had it been merely a phase of fashion or of eccentricity, would long since have been buried in oblivion, or would, at best, be dying a lingering death. The Jaeger system is believed, rightly or wrongly, to be endowed with great personal interest for each one of us, as a means for the preservation of health, and even of life.

Dr. Jaeger claims no less than this for his reforms, in his shilling volume, entitled "Health Culture" (Waterlow and Sons), which certainly contains a great deal of new and interesting matter that renders the book well worth procuring and reading carefully through. Speaking for ourselves, while, of course, not endorsing unreservedly Dr. Jaeger's writings, we find very little to contest in the arguments set forth in favour of all-wool clothing and bedding. The leading principle—that the skin should have full freedom of action—*i.e.*, that it should be able to throw off the watery vapour which is constantly exhaled through the pores—is undoubtedly correct; and although this must have been clear to every physiologist who has considered the matter, the fact remains that until Dr. Jaeger urged this important principle on the general attention, pointing out that ordinary clothing and bedding completely violate it, the hygienic considerations involved had been generally ignored.

Another valuable contribution by Dr. Jaeger to the science of covering the human body, consists in his demonstration of the respective merits of animal and plant fibre as materials for such covering. Dr. Jaeger's conclusions as to the unsuitableness of plant-fibre (linen and cotton) have not, so far as we are aware, been either anticipated or attempted to be refuted; and he is entitled to the merit of an original discovery when he shows that plant fibre being impervious to the skin's exhalation, is chilling, because a ready heat conductor. Of course we know that living plants absorb the carbonic acid and other impurities in the air, but these matters are assimilated by plants, while the dead plant-fibre gives them out again when warmed or wetted. Hence, according to Dr. Jaeger, clothing and bedding of plant-fibre attract and store up the noxious matters which are given off from the skin and lungs of human beings; and when the temperature rises or the air is damp, these materials give forth their poisonous accumulations, which are then re-inhaled by the wearer or sleeper.

On the other hand, animal fibre, which is of a horny character, and has been devised by Nature as clothing for an animal body, takes up no noxious matters from the atmosphere, the exhalation from the skin passing freely away through it, when woven into fabric of porous web.

Dr. Jaeger argues that when, by means of pervious clothing and bedding (*i.e.*, of animal wool throughout, with no admixture of plant-fibre in any shape), the skin is enabled to perform its vital function freely, the body is promptly relieved of the noxious matter constantly excreted through the pores, and the tissues are drained and hardened. At the same time the skin is maintained at an equable normal temperature; for the animal wool covering, with its slow-heat-conducting quality, protects from chill, while so soon as the internal temperature of the body reaches a point at which oppressive heat is felt, the pores act freely through their pervious covering, and the body is cooled in a safe and natural manner.

Many further striking and novel considerations are presented in Dr. Jaeger's "Health Culture," which we have not space to detail here, but which our readers may readily learn for themselves on reference to his book.

A very satisfactory feature of Dr. Jaeger's reforms is the practical manner in which he has made it possible for all who desire to do so to adopt them. Not satisfied with laying down rules which the public at large would find difficult to carry out, meeting, as was inevitable at first, with the passive resistance of makers and vendors of clothing on the old principles, Dr. Jaeger induced certain manufacturers to produce first one and then another article after his own ideas, until now the reform of every item of clothing and bedding, for men, women, and children, is an accomplished fact; and doubtless the popularity which the Jaeger system has attained is largely due to the complete manner in which it is presented at the depôts of the Jaeger Company (Limited), at 41 to 44, Fore Street (close to Moorgate Street Station), and 3 and 4, Princes Street, Cavendish Square, where descriptive price lists can be obtained.

